# Self-Healing Composite Coating Fabricated with a Cystamine Cross-Linked Cellulose Nanocrystal-Stabilized Pickering Emulsion

**DOI:** 10.1021/acs.biomac.3c00915

**Published:** 2024-01-25

**Authors:** Guofan Xu, Amaka J. Onyianta, Jean-Charles Eloi, Robert L. Harniman, Jude Laverock, Ian Bond, Onajite Abafe Diejomaoh, Todor T. Koev, Yaroslav Z. Khimyak, Stephen J. Eichhorn

**Affiliations:** † Bristol Composites Institute, School of Civil, Aerospace and Design Engineering (CADE), University of Bristol, University Walk, Bristol BS8 1TR, U.K.; ‡ School of Chemistry, University of Bristol, Bristol BS8 1TS, U.K.; § School of Pharmacy, University of East Anglia, Norwich Research Park NR4 7TJ, U.K.

## Abstract

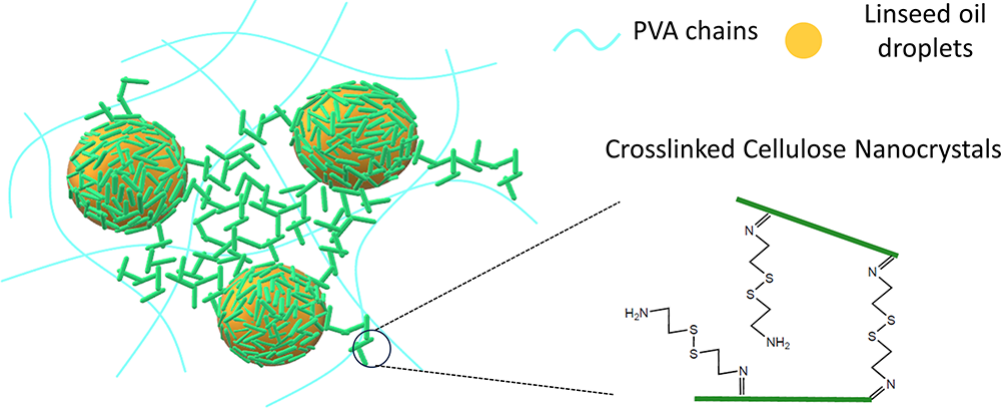

A gelled Pickering emulsion system was fabricated by first stabilizing linseed oil droplets in water with dialdehyde cellulose nanocrystals (DACNCs) and then cross-linking with cystamine. Cross-linking of the DACNCs was shown to occur by a reaction between the amine groups on cystamine and the aldehyde groups on the CNCs, causing gelation of the nanocellulose suspension. Fourier transform infrared spectroscopy and X-ray photoelectron spectroscopy were used to characterize the cystamine-cross-linked CNCs (cysCNCs), demonstrating their presence. Transmission electron microscopy images evidenced that cross-linking between cysCNCs took place. This cross-linking was utilized in a linseed oil-in-water Pickering emulsion system, creating a novel gelled Pickering emulsion system. The rheological properties of both DACNC suspensions and nanocellulose-stabilized Pickering emulsions were monitored during the cross-linking reaction. Dynamic light scattering and confocal laser scanning microscopy (CLSM) of the Pickering emulsion before gelling imaged CNC-stabilized oil droplets along with isolated CNC rods and CNC clusters, which had not been adsorbed to the oil droplet surfaces. Atomic force microscopy imaging of the air-dried gelled Pickering emulsion also demonstrated the presence of free CNCs alongside the oil droplets and the cross-linked CNC network directly at the oil–water interface on the oil droplet surfaces. Finally, these gelled Pickering emulsions were mixed with poly(vinyl alcohol) solutions and fabricated into self-healing composite coating systems. These self-healing composite coatings were then scratched and viewed under both an optical microscope and a scanning electron microscope before and after self-healing. The linseed oil was demonstrated to leak into the scratches, healing the gap automatically and giving a practical approach for a variety of potential applications.

## Introduction

Pickering emulsions, first researched by Ramsden^1^ and later named after the pioneering work done by Spencer Umfreville Pickering, is a special type of emulsion system in which solid particles are used as the stabilizer.^2^ Solid particles satisfying the partial wetting condition are irreversibly adsorbed to the oil–water interface, stabilizing the emulsions.^3−5^ Different types of particles have been utilized to fabricate Pickering emulsions, e.g., latex particles,^3,6−8^ silicates or silica,^9−13^ graphene oxide,^14−18^ protein particles,^19−22^ and cellulose nanomaterials (CNMs).^23−28^ Oil droplets in Pickering emulsions stabilized with CNMS tend to cream because of the interactions between the CNMS and their low density.^23,29−31^ Leaking of oil occurs in the creaming layer after the aggregation and close packing of oil droplets.^32^ The creaming and the following oil leaks can be prevented by adding additional surfactants or synergistic chemicals or additives to stabilize the emulsion^26,27,33^ or by the covalent modification of CNMs.^7,24,25,31,34^

Linseed oil is a natural oil that can automatically dry and harden upon oxidation in air.^35,36^ It has been used as a healing agent in self-healing composites by encapsulating the oil in emulsions or Pickering emulsions.^31,37−41^ Urea–formaldehyde (UF) capsules have been synthesized in previous research with linseed oil inside, mixed with epoxy resin, and then fabricated into corrosion-inhibiting coatings on metal substrates.^37−39,41^ The use of cellulosic materials acting as emulsifiers and encapsulating linseed oil in Pickering emulsions has been reported.^31,40^ Self-healing gels have been made with linseed oil by encapsulating the oil with CNMs in water and then polymerizing the emulsion into a hydrogel by adding glycerol.^40^ A self-healing coating has also been previously fabricated with linseed oil by mixing cellulose-stabilized linseed oil-in-water Pickering emulsions with a waterborne varnish.^31^

Within the glucose repeat units of cellulose, the C2–C3 bonds can be broken by periodate oxidation, forming 2,3-dialdehyde groups.^42−45^ The resulting product is commonly referred to as dialdehyde cellulose (DAC), and the aldehyde groups can be further transformed into carboxylic groups,^46−51^ primary alcohols,^52,53^ or imine groups.^31,54−66^ Imine groups are formed through the Schiff base reaction between the aldehyde groups on DACs and the amine groups on the other reactants. These imine groups and the resulting Schiff base can also be reduced to new amines.^31,57,60,62,64,66^ Cystamine has been used to cross-link DAC materials by forming Schiff bases with the two amino groups on its two ends.^61,67−69^ The resulting DAC hydrogels were demonstrated to be redox- and pH stimuli-responsive^61,65,67,69^ and have demonstrated self-healing abilities.^65,68^ All the previous work focused on the properties of cysCNC hydrogels; however, research on the properties of dried cysCNC aerogels or the application of cysCNCs in Pickering emulsions is still lacking.

In this work, we report the formation of self-healing composite coatings fabricated by combining gelled Pickering emulsions with a PVA solution. The Pickering emulsions were prepared from the mixtures of linseed oil and dialdehyde cellulose nanocrystals (DACNCs) cross-linked with cystamine. The presence of cystamine promoted cross-links of the DACNCs throughout the Pickering emulsion, both at the oil–water interface and in the continuous water phase, resulting in a gelled Pickering emulsion without creaming or aggregation of oil droplets. Cross-linking is monitored through changes in the viscoelastic rheological profile of the Pickering emulsions. PVA, as a popular water-based synthetic polymer, was chosen to be the matrix for the final composite coating, demonstrating the capability of the cross-linked Pickering emulsion system with water-based polymers. Combining PVA with CNC and linseed oil finally resulted in a biodegradable and sustainable self-healing system.

## Materials and Experimental Methods

### Materials

Linseed oil (yellow liquid, flash point 113 °C, density 0.93 g cm^–3^ at 25 °C) was purchased from Merck Life Science UK Ltd. (Dorset, UK). Extra pure ethylene glycol 99+% was purchased from Thermo Fisher Scientific (Lancashire, UK). Poly(vinyl alcohol) (PVA, Mw 89,000–98,000, 99+% hydrolyzed), cystamine dihydrochloride, dichloromethane (DCM), and solid potassium periodate 99.8% (230.00 g/mol) were purchased from Merck Life Science UK Ltd. (Dorset, UK). Freeze-dried CNCs (sodium form) with a 0.94 wt % sulfur content were purchased from the Process Development Center, University of Maine (Maine, USA). Dowex Marathon C hydrogen form strong acid cation (SAC) exchange resin was purchased from Merck Life Science UK Ltd. (Dorset, UK).

### Chemical Modification of CNCs

**Scheme 1:**
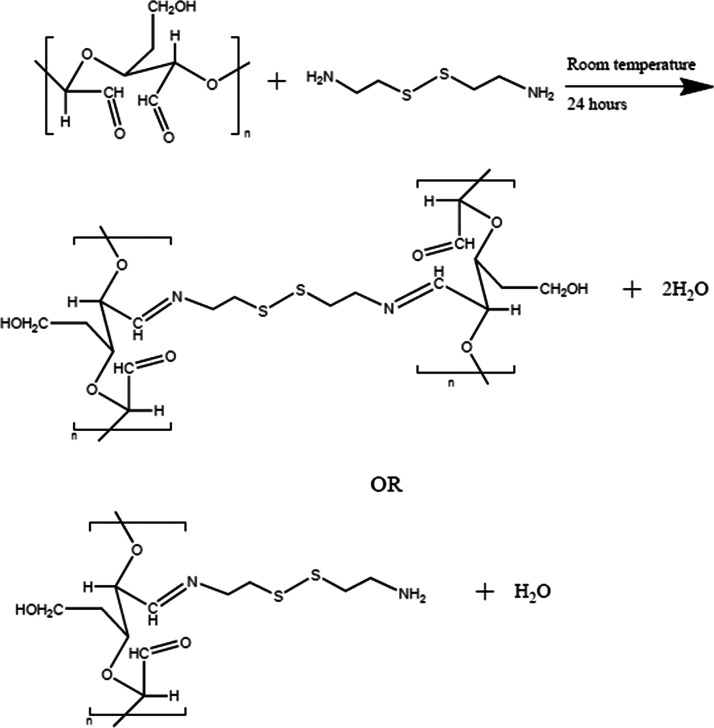
Two-Step Schiff Base Reaction Pathway from sCNC to cysCNC

Freeze-dried and sulfated CNCs were modified with cystamine through periodate oxidation and a Schiff base reaction (Scheme 1).^30,60^ Sulfated CNC aqueous suspensions (1.5 wt %) were first made by dispersing the nanocrystals in DI water using a sonic probe (Branson Digital Sonifier). This sCNC suspension was then reacted with 1.68 mmol of sodium periodate per 1 g of CNCs. After reacting for 48 h, the DACNC suspension was dialyzed against DI water using a cellulose membrane (molecular weight cutoff of 14 kDa) for over 48 h. Part of the purified DACNCs were stored in the fridge in glass bottles and then reacted with cystamine for further modification. The pH of the DACNC suspension was kept within the range of 4–5 to provide an optional reaction condition for the Schiff base reaction. Cystamine, which had been neutralized by adding NaOH to cystamine dihydrochloride aqueous solution and extracted with DCM by solvent exchange and solvent evaporation, was placed dropwise into the DACNCs (4 mmol per 1 g of CNCs) and dispersed with a magnetic stirrer. After 10 min of stirring, the mixture was left unperturbed for the Schiff base reaction to occur at room temperature. After reacting for 24 h, the cystamine-CNCs(cysCNCs) were washed with 2 w/v % NaCl in the water/isopropanol mixture (50/50 v/v) and freeze-dried for further characterization. The reaction pathway is shown in Scheme 1.

### Preparation of Gelled Pickering Emulsions

Linseed oil was dropped into purified DACNC aqueous suspensions of different concentrations (0.5, 1, 2, 3 wt %), with a CNC:oil ratio of 20%. A sonic probe was then used to break the continuous oil phase into small oil droplets that were uniformly dispersed in the DACNC suspension. DACNCs were automatically adsorbed at the oil–water interface, thereby stabilizing the resulting Pickering emulsions. Cystamine was then added to this Pickering emulsion and stirred with a magnetic stirrer for 10 min. The Pickering emulsion with cystamine was then left for the Schiff base reaction to occur at room temperature for 24 h, resulting in what we term a gelled Pickering emulsion system.

### Fourier Transform Infrared (FTIR) Spectroscopy

FTIR spectroscopy (Spectrum 100, PerkinElmer, USA) was used to distinguish sCNCs, DACNCs, and cysCNCs. A small portion of each form of the CNC gels was thoroughly washed and freeze-dried with a FreeZone 2.5 L −84C Benchtop Freeze-Dryer (Labconco Corporation) to obtain the required solid samples. The absorbance of the IR signals (600–4000 cm^–1^) was normalized using a band located at 1030 cm^–1^ for all the CNC samples and compared with cystamine dihydrochloride.

### ^1^H–^13^C Cross-Polarization Magic Angle Spinning Nuclear Magnetic Resonance (CP/MAS NMR) Spectroscopy

Solid-state NMR experiments were performed on a Bruker Avance III NMR spectrometer equipped with a 4 mm triple resonance probe operating at frequencies of 400.22 MHz for ^1^H and 100.63 MHz for ^13^C. The cysCNC powder sample was packed tightly into an 80 μL rotor and spinned at an MAS rate of 6 kHz. The ^1^H–^13^C CP/MAS NMR spectrum was recorded at 20 °C using 5000 scans, a recycle delay of 10 s, and a contact time of 2 ms.

### ^1^H Solution-State and Diffusion-Ordered Spectroscopy (DOSY)

cysCNC (*ca*. 10 mg) was dissolved in DMSO-*d*_6_, and the ^1^H spectrum was acquired at 20 °C, 64 scans, and a recycle delay of 4 s. The self-diffusion coefficients of the CNC backbone (ca. 3.2–4.0 ppm) and the cystamine moieties (ca. 2.8–3.0 ppm) were measured using a stimulated echo sequence with bipolar gradients (Bruker’s *stebpgp1s*). The peaks’ intensity was recorded as a function of 16 different gradient strength values (2–98%), obtaining >98% signal attenuation. All experiments were acquired using 8 scans per gradient strength value, an acquisition time of 1.4 s, a relaxation delay of 4 s, a diffusion duration of 0.16 s, a gradient pulse length of 7 ms, and a gradient recovery delay of 200 μs. The peaks of interest were integrated, and their signal decay as a function of gradient strength was fitted (eq S1) using Bruker’s Dynamics Centre to obtain an average of the CNCs’ and cystamine’s self-diffusion coefficients.

### Conductivity Titration

The average surface charge densities of cellulose nanocrystals (CNCs) with sulfate half ester charge groups (both sCNCs and DACNCs) were measured through conductivity titration following a CNC surface charge titration protocol.^70^ For freeze-dried sodium-form sCNCs, the samples were redispersed in water with a 0.5 wt % concentration and dialyzed against DI water for 3 days to remove any free sodium ions. The suspension was then poured into a column filled with Dowex Marathon C hydrogen form strong acid cation (SAC) exchange resin and a fritted glass disk at the bottom. The sodium ions on the sCNC surfaces are exchanged with protons when the suspension passes through the SAC resin. The resulting acid-form sCNC suspension was then titrated with NaOH to get the mean sulfate half-ester content on the CNC surface. DACNC suspensions were also protonated with SAC resin before titration with the same method. All of the titration experiments were performed in triplicate to get the average data and the standard error of the mean.

### X-ray Photoelectron Spectroscopy (XPS)

XPS with a monochromatic Al Kα X-ray source (1486.7 eV) was utilized to detect sulfur on the cysCNCs. Sulfur from both disulfide bonds in grafted cystamine groups and sulfate half-ester groups on the original sCNCs was distinguished in this XPS analysis. CysCNCs were drop-cast onto Mo foils with multiple layers to increase the density of the cysCNC and were subsequently delaminated from the foil and loaded onto carbon tape for analysis. Before measuring, the CNC samples were outgassed under ultrahigh vacuum for over 24 h. Spectra were acquired by using pass energies of 50 eV for survey scans and 20 eV for high-resolution measurements. A charge neutralizer (operating at a beam energy of 4.5 eV and an electron flux of 3 μA) was used to mitigate sample charging during the X-ray measurement. The energy axis was charge referenced to adventitious carbon at 284.8 eV. To estimate relative concentrations of sulfur components, the background was subtracted using a Shirley-type background model, and the sulfur peaks were fitted using a combination of two doublets with Voigt lineshapes.

### Thermogravimetric Analysis (TGA)

Before the TGA test, all types of CNC suspensions were freeze-dried into “fluffy” solid materials. These freeze-dried CNCs were then tested in nitrogen within the temperature range of 30–600 °C at a heating rate of 10 °C min^–1^. Derivative thermogravimetric curves were obtained by performing a first derivative on the percentage weight loss data from TGA using Origin software (version 2021b).

### Transmission Electron Microscopy (TEM)

TEM was used to characterize the dimensions, as well as aggregation properties of DACNCs and cysCNCs. Drop-cast suspensions of DACNC and cysCNC (1 mg/mL) onto glow-discharged (20s plasma in an oxygen-containing low-pressure atmosphere using a Q150TES from Quorum Technologies Ltd, UK) carbon-coated grids, immediately stained with a 2 wt % uranyl acetate solution, were imaged using a 200 kV field-emission gun transmission electron microscope (JEM-2100F, from JEOL, Japan), equipped with an Orius SC1000 camera, from Gatan (now AMETEK), USA.

### Water Contact Angle (WCA) Test

A DSA100 Drop Shape Analyzer (KRÜSS) was used to measure the static contact angle of DI water on sCNC, DACNC, and cysCNC films. All the CNC films were fabricated by drop-casting CNC suspensions (1.5 wt %) on glass slides. An ultrathin needle was used to drop DI water onto the films. Multiple drops were made on each film to avoid the influence of surface imperfections on the water contact angles.

### Rheological Testing of cysCNC Gels and Gelled Pickering Emulsions

The effects of the Schiff base reaction between cystamine and DACNCs on the viscoelastic properties of the CNC suspensions and the DACNC-stabilized Pickering emulsions were studied by using a 40 mm serrated Peltier parallel plate geometry within a DHR rheometer (TA Instruments, USA). The DACNC suspensions and DACNC-stabilized Pickering emulsions with CNC concentrations 0.5, 1, 2, and 3 wt % were mixed with cystamine and then loaded on the serrated Peltier plate with a moisture cover for 24 h oscillation time sweep at strain 0.1% with a frequency of 1.0 Hz and were gelled at room temperature. The viscoelastic properties of cysCNC gels of 1, 2, and 3 wt % were also measured through an oscillation frequency sweep (angular frequency from 0.5 to 50 rad/s) at a strain of 0.5% on the serrated Peltier plate and compared with a 4 wt % sCNC suspension. The viscoelastic properties of sCNC suspensions with solid contents lower than 4 wt % cannot be measured on the DHR rheometer.

### Dynamic Light Scattering (DLS) Test of DACNC-Stabilized Pickering Emulsion and DACNC Suspension

The size of the oil droplets in the DACNC-stabilized Pickering emulsions before gelling was measured using a Zetasizer Nano S90 particle analyzer (Malvern Instruments Ltd.). The instrument uses a 4 mW 632.8 nm “red” laser and a 90° scattering detector angle for size measurement. An option of “Multiple Narrow Modes” in the instrument was used for the testing of the Pickering emulsions as the samples were thought to contain both oil droplets and CNC rods and clusters, which would give a distribution of several narrow peaks. Intensity size distributions of the particles were obtained from the measured correlation function by Zetasizer software using algorithms extracting the decay rates for a series of size classes. DACNC suspensions with the same CNC concentration as the Pickering emulsions were used as the control group. It is noted that size scales quoted for the DLS data are diameter and so mostly refer to an equivalent sphere of that dimension.

### Confocal Laser Scanning Microscopy (CLSM) of DACNC-Stabilized Pickering Emulsion

DACNCs and linseed oil were first stained with Calcofluor White and Nile Red, respectively, before preparing Pickering emulsions. These Pickering emulsions were then viewed with a Leica SP5-II confocal laser scanning microscope, which was attached to a Leica DMI 6000 inverted epifluorescence microscope. A solid-state 20 mW 561 nm laser and a 50 mW 405 nm laser were used for the experiment. UV radiation was utilized as the fluorescence excitation of Calcofluor White, and green light was utilized to excite the fluorescence of Nile Red. Two separate channels, one in the range 400–500 nm and another in the range 600–700 nm, were set to obtain fluorescence emission from Calcofluor White and Nile Red, respectively.

### Calculation of the Mass of CNCs Adsorbed on the Oil Droplet Surface

The mean CNC shell thickness can be predicted with the assumption that the CNCs at the water–oil interface comprise a shell around the oil droplets with a thickness (*x*) according to the equation^71^
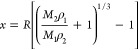
1where *R* is the mean core radius of the oil droplets, *M*_1_ and *M*_2_ are the mass fractions of the core and shell components, and ρ_1_ and ρ_2_ are the densities of the core and shell components, respectively. If it is assumed that all the CNCs in the water suspension adsorb to the water–oil interfaces and form the CNC shells around the oil droplets (knowing *M*_2_/*M*_1_), then the CNC shell thickness *x* can be predicted. Also, if the CNC shell thickness is known, then the mass fraction ratio of the shell and core material can be calculated using a rearrangement of eq 1 as follows.
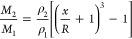
2

### Atomic Force Microscopy (AFM)

AFM was conducted utilizing a multimode VIII microscope with a Nanoscope V controller (Bruker, CA, USA). The combination of a Fastscan unit with PeakForce feedback control enabled regions to be investigated multiple times at different force levels applied between the cantilever tip and the sample. With SCANASYST-AIR-HR cantilevers of nominal tip radius and spring constant 2 nm and 0.4 N/m, respectively, tip–sample forces in the range of 600 pN–18 nN were applied. In this way, the surface and subsurface structure of oil droplets could be imaged. Samples were prepared through the drop-casting of 5 μL of emulsion onto cleaned silicon substrates and investigated in an ambient environment.

### Scanning Electron Microscopy (SEM) of Freeze-Dried Gelled Pickering Emulsions

Gelled Pickering emulsions (5 mL) were frozen in a plastic tube with liquid nitrogen and lyophilized in a FreeZone 2.5 L −84C Benchtop Freeze-Dryer for 3 days. The freeze-dried gelled Pickering emulsion sample was then cut into thin slices with a scalpel, secured on a carbon sticky pad, and coated with a thin layer (∼10 nm) of high-purity (99.99%) Ag with a high-resolution sputter coater (Agar Scientific, UK) before being imaged by SEM. SEM micrographs were obtained on a JSM-IT300 (JEOL, Japan) system operated at an accelerating voltage of 15 kV at a working distance of 10.5 mm, detecting secondary and backscattered electrons.

### Self-healing PVA Coating Preparation

**Scheme 2:**
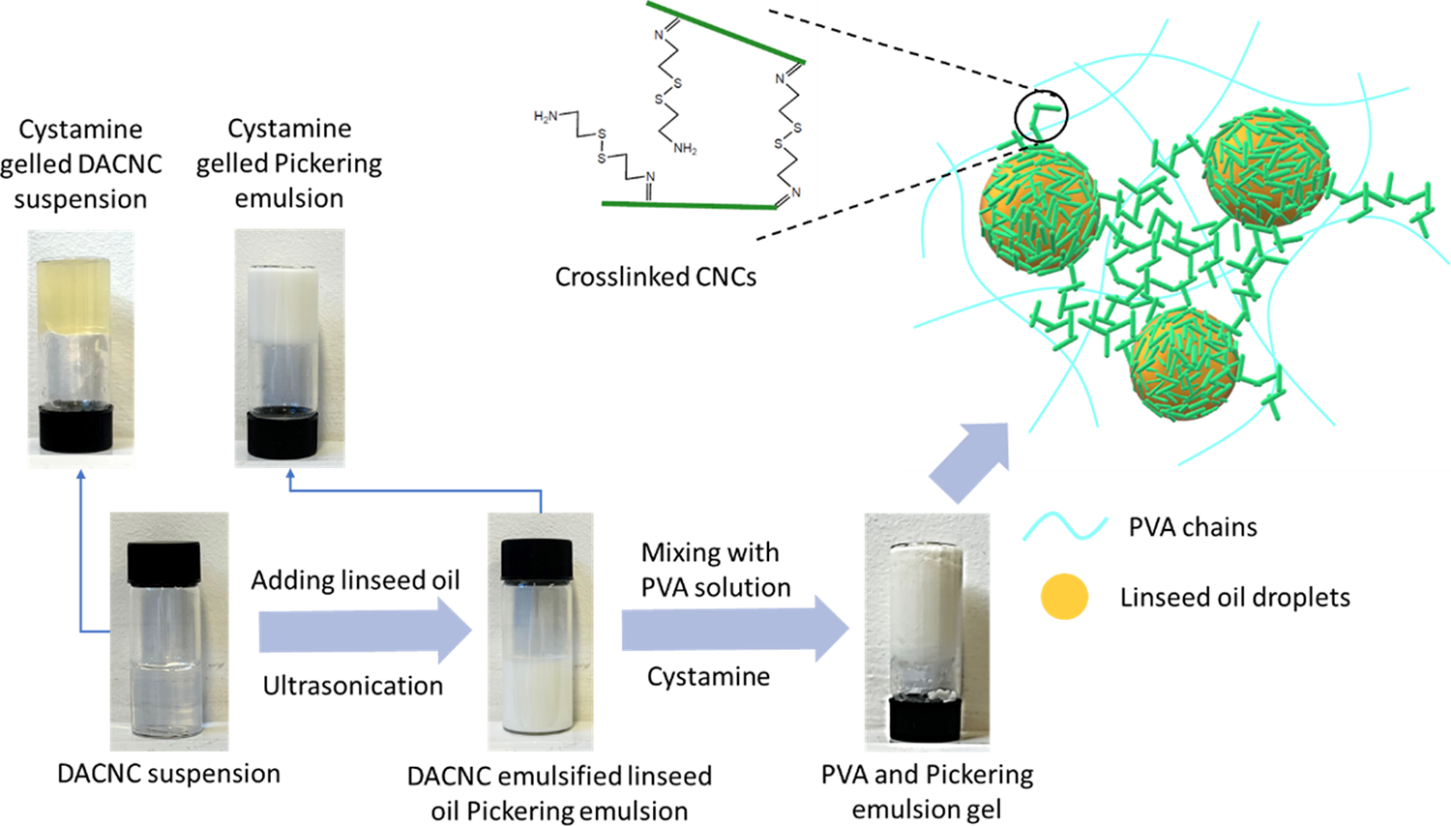
Fabrication Pathway of Gelled PVA and the DACNC-Stabilized Pickering Emulsion Mixture It is noted that fully cross-linked CNCs via imide bonds may not be present in the system (see reaction Scheme 1).

PVA solution (10%) was first prepared by dissolving 10 g of PVA powder with 100 mL of DI water at 90 °C for 3 h under magnetic stirring. This PVA solution was then mixed with DACNC-stabilized Pickering emulsions using magnetic stirring and a sonic bath, keeping the solid weight of PVA the same as the weight of added linseed oil. Cystamine was then inserted into the PVA and Pickering emulsion mixtures and stirred for 10 min. The mixture was then kept at room temperature for cross-linking to take place. After the reaction, the mixture was drop-cast onto glass slides and gradually dried into coatings at room temperature. The fabrication of the gelled PVA and DACNC-stabilized Pickering emulsion system is shown in Scheme 2.

### Scratch and Self-Healing of the PVA Coating

The self-healing ability of PVA coatings with cysCNC-stabilized linseed oil droplets was tested by scratching the coating with a scalpel, which was then left to heal automatically. The scratched coatings were heated to 80 °C in the oven and stored at this temperature for 16 h to accelerate the oxidation of leaked linseed oil in the scratched areas. A room-temperature healing test was carried out by scratching the coating and leaving it at room temperature without any heating for 21 days. The coatings with scratches were viewed under SEM before and after healing without any conductive medium in low-pressure vacuum mode.

## Results and Discussion

### Physical Characterization of cysCNCs

**Figure 1:**
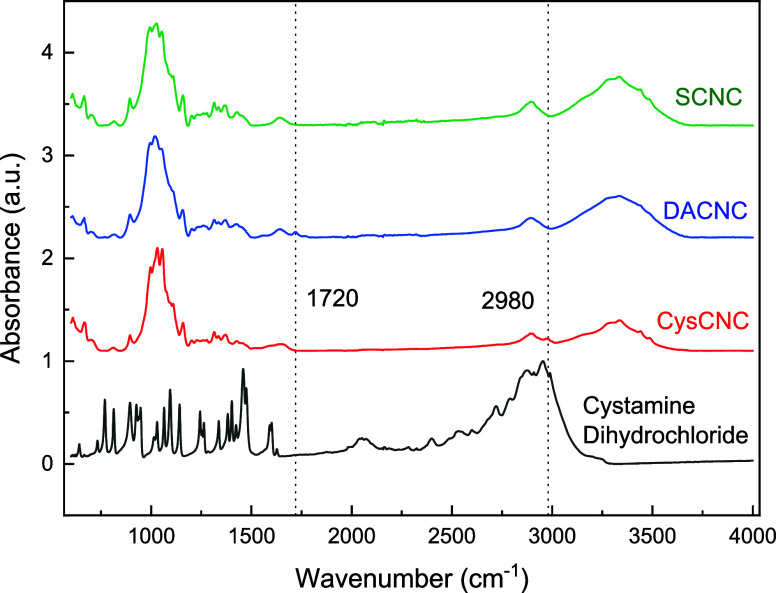
Typical FTIR spectra for sCNC, DACNC, cysCNC, and cystamine dihydrochloride. a.u.— arbitrary units.

The infrared absorbance spectra for CNCs and cystamine dihydrochloride are shown in Figure 1, both demonstrating the presence of cystamine groups on the cysCNCs. The IR absorption of C=O stretching is observed at a wavenumber position of 1720 cm^–1^ for DACNCs, which was not present for both sCNCs and cysCNCs. It is thought that the disappearance of the C=O band in cysCNCs was caused by the Schiff base reaction occurring between dialdehyde groups on CNCs and amine groups on cystamine. An absorbance band located at ∼2980 cm^–1^ was detected for properly washed and freeze-dried cysCNCs, which was not observed for either sCNCs or DACNCs. The presence of this band indicates that additional −CH_2_ stretching was detected on the cysCNCs.^72,73^ The infrared absorbance of cystamine dihydrochloride (solid) was also analyzed and compared with that of cysCNCs as a reference. The cystamine dihydrochloride exhibited absorbance bands in the range 2800–3000 cm^–1^, which is attributed to −CH_2_ stretching. According to the conductivity titration experiments (Figure S1), the average surface charge densities of sCNCs and DACNCs with sulfate half ester groups were found to be 316 ± 9.6 and 311 ± 14.7 mmol/kg, respectively (the errors provided are standard errors from the mean) through conductivity titration.

The cysCNC samples were also analyzed by using XPS to further demonstrate the presence of cystamine groups (Figure S2). Sulfur was found to be present in both sulfate half-ester groups and the disulfide bonds of the cystamine groups. Multiple layers of cysCNCs were drop-cast on the Mo foil to increase the intensity of the signals from both forms of sulfur in the XPS. Elements O, C, N, and S were detected in the XPS spectra, and two S 2p peaks were observed, one at 164.0 eV due to the sulfide environment and another at 168.8 eV due to the sulfate environment (Figure S2). After fitting the intensities for sulfur and performing a calculation, we found that approximately 58% of the sulfur on cysCNCs was found in sulfide (disulfide bond in the cystamine group) for the samples on C tape, and the remaining 42% of sulfur was found in the sulfate environment (sulfate half-ester group). These two environments for sulfur were also observed at the Sulfur 2s level. During the experiment, the sulfide peak was found to decrease after extended exposure to the X-rays, which indicated that the S–S bonds can be broken by radiation.^74^

**Figure 2:**
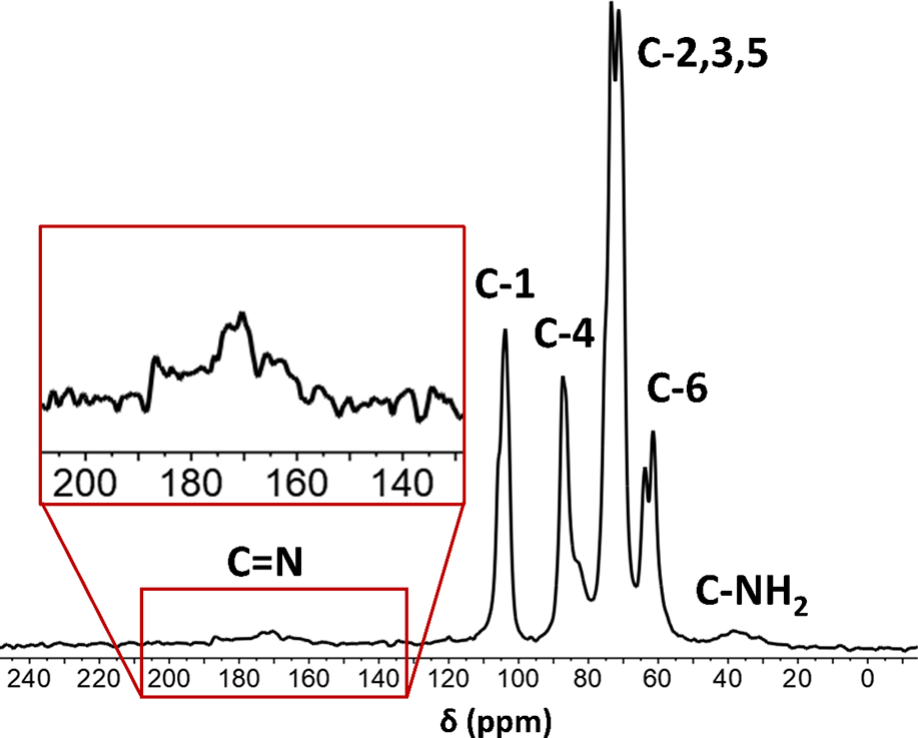
^1^H–^13^C CP/MAS NMR spectrum of cysCNC, featuring a CNC monomer peak assignment. Inlay showing the imine peak at ca. 172 ppm.

The solid-state ^1^H–^13^C CP/MAS NMR spectrum (Figure 2) of cysCNC showed the presence of a peak at ∼172 ppm in the sample characteristic of an imine bond (C=N), proving the successful modification of DACNC with cystamine. The ^1^H–^13^C CP/MAS NMR spectrum also showed the presence of an amine bond (C–NH_2_, ∼37 ppm), likely due to a proportion of free cystamine residues or them only being covalently bonded to a CNC on one side (i.e., one imine and one amine). Upon solubilization, noncovalently bound cystamine species are present in the solution. Hence, the ^1^H solution-state and DOSY NMR spectra of the solubilized material (Figures S3 and S4), and self-diffusion coefficient fittings showed several orders of magnitude faster self-diffusion coefficients for the cystamine moiety (7.16 × 10^–10^ m^2^/s) compared to CNC (4.14 × 10^–13^ m^2^/s). The simulated self-diffusion coefficient^75^ of free cystamine in DMSO-*d*_6_ at 20 °C is ca. 3.6 × 10^–10^ m^2^/s–similar to the experimental values for the cystamine moieties in the cysCNC sample. Together, these data suggest the final product of the Schiff-type reaction being a combination of cysCNC – both single- and double-bridged as well as cystamine adsorbed on the surface of CNC.

The thermal stabilities of all samples were characterized by using the TGA test, the results of which are presented in Figure S5. The onset degradation temperature for DACNCs and cysCNCs was found to be ∼250 °C, which is about 50 °C lower than that for the sCNCs. We suggest that this decrease in the onset degradation temperature is caused by the breaking of the C2–C3 bonds in the cellulose backbone chains. The degradation rate for both DACNCs and cysCNCs was found to be slower than that for the sCNC samples. The optimum degradation temperatures (OPT) for the cysCNC and sCNC appeared to exhibit no significant difference, while the OPT for DACNC appeared to shift to a higher value by ∼20 °C. In cystamine groups’ cross-linking, all the DACNCs seemed to decelerate the degradation of CNC, and the cysCNCs were thermally stable enough to be heated up until about 250 °C, which would allow the material to be used in heated self-healing systems.

**Figure 3:**
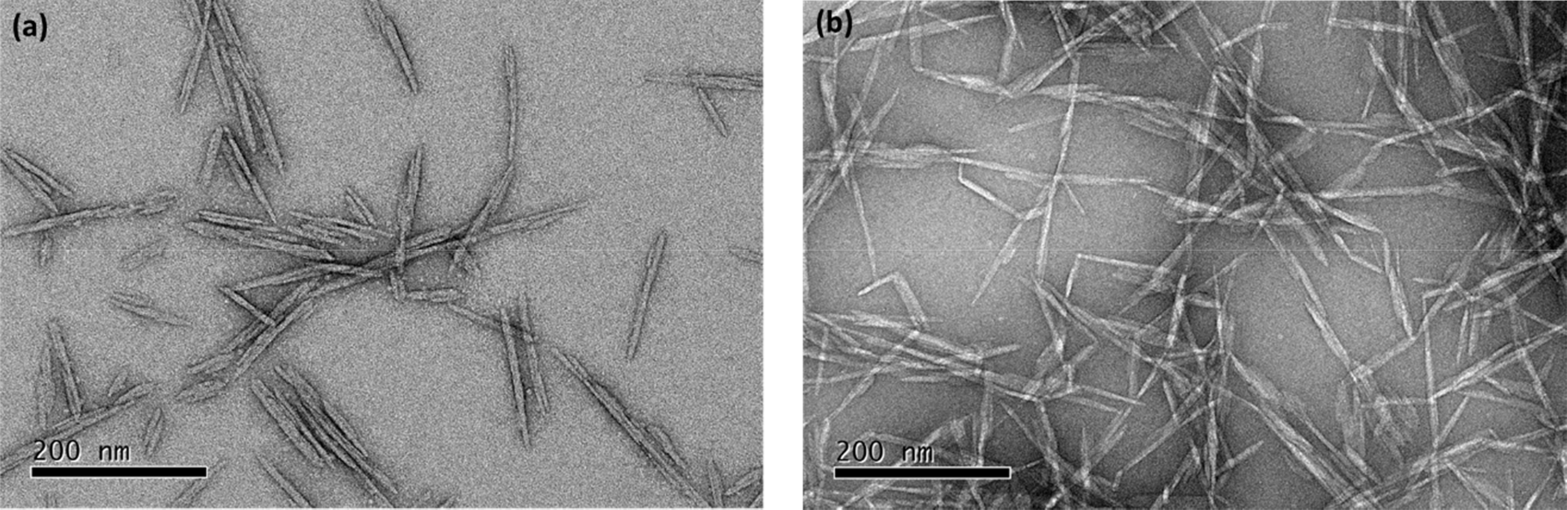
Typical TEM images of DACNC (a) and cysCNC (b). Scale bars are 200 nm. All samples were imaged by using the same initial concentration in solution.

**Figure 4:**
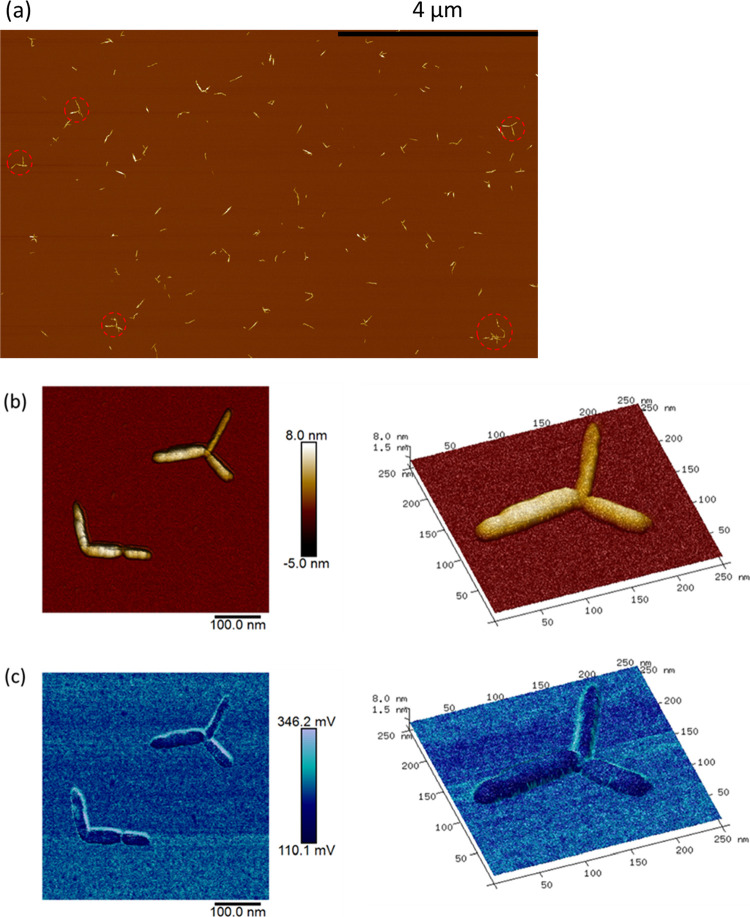
AFM height image of 0.005 wt % cysCNC droplets deposited and air-dried on silicon (a) and zoomed-in height image (b) and adhesion image (c) of linked CNC clusters.

Representative bright-field TEM images for stained DACNCs and cysCNCs are shown in Figure 3. Interconnections among CNCs and aggregation were observed for both samples at equal concentrations. However, for the DACNC samples, both CNC clusters and isolated CNC rods were detected, and little interconnection between CNC clusters was observed. The cysCNCs were found to be completely interconnected and cross-linked into a network, on some occasions side-to-side, but mostly tip-to-tip, even at a low concentration (1 mg mL^–1^) (more TEM images in Figure S6). This tip-to-tip linking among cysCNCs was also detected in the AFM of cysCNCs deposited on silicon (Figure 4). This cross-linked network presumably arises through the formation of imine groups from the presence of cystamine. The DACNCs were measured to have average lengths and widths of 113.7 ± 4.9 and 8.0 ± 0.2 nm, respectively, similar to the cysCNCs’ average size which was measured to be 111.8 ± 6.5 nm in length and 8.2 ± 0.2 nm in width. All the errors presented here are standard errors from the mean, and the standard deviation of each datum is shown in Table S1.

The AFM cysCNC sample of 0.05 wt % was allowed to deposit for 30 s before being rapidly dried with a nitrogen gas flow. This seemed to have resulted in a good dispersion of CNCs to work with. In the scan of deposited sycCNCs, various star-shaped aggregates were observed, which suggest end-to-end attachment among CNCs (red circled in Figure 4a), something that has previously been observed, albeit for a different form of modification.^76^ In the scans of linked CNCs (Figure 4b,c), particularly, the example of a three-linked tip-to-tip, it was clear that materials linking the CNCs are lower in height than the CNCs themselves. It is thought that end modification of the CNCs contributes to this tip–tip assembly mechanism.

**Figure 5:**
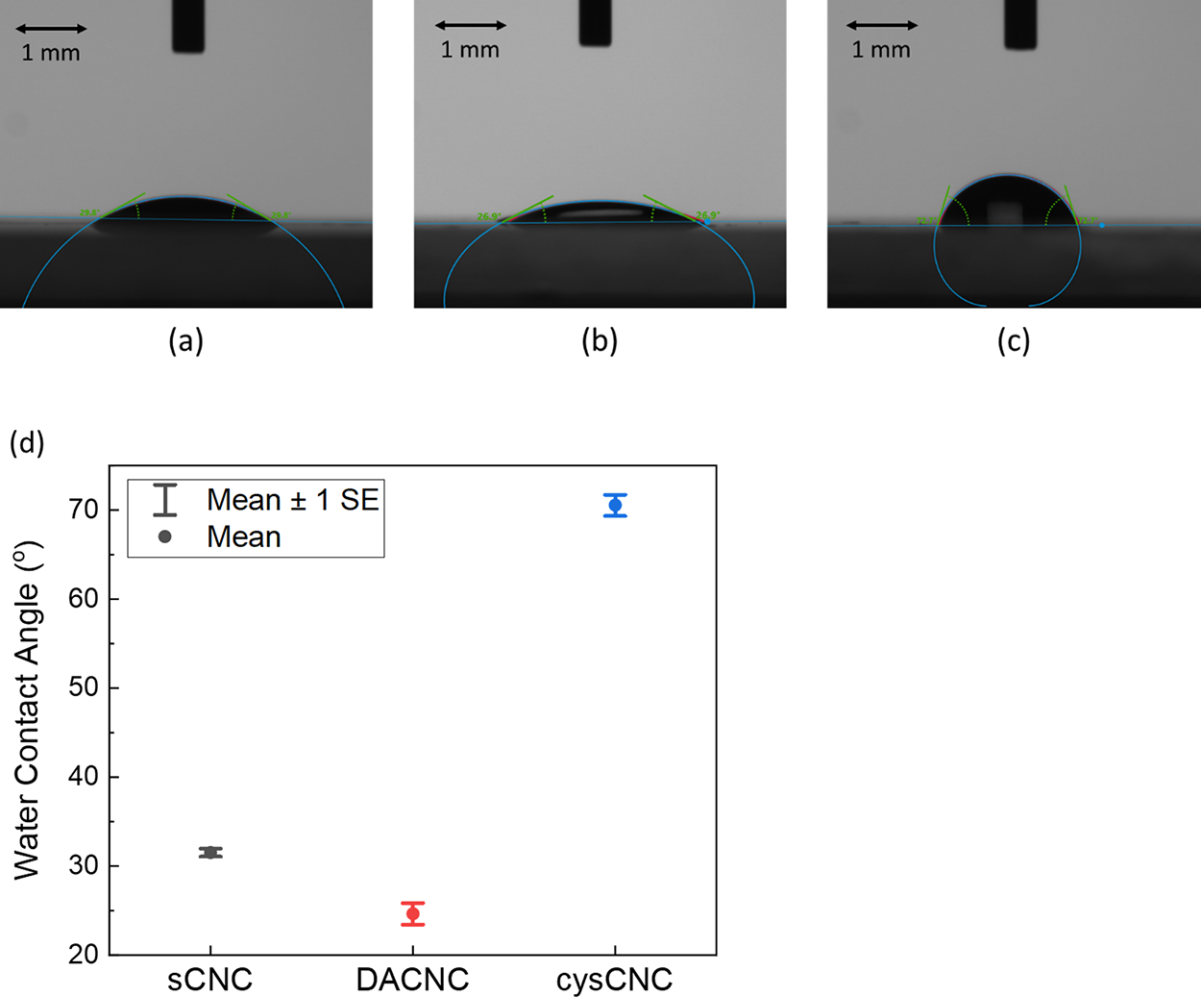
Typical photos of DI water droplets on sCNC (a), DACNC (b), and cysCNC (c) and the average water contact angle of CNCs (d) (*p* < 0.05, *n* = 10).

The hydrophobicity of the CNCs was demonstrated by dropping DI water onto drop-casted sCNC, DACNC, and cysCNC films on glass slides. Water contact angles for these three kinds of CNCs were measured. Images of typical water droplets on CNC films are shown in Figure 5. The water contact angles for sCNC, DACNC, and cysCNC were found to be 31.2 ± 0.8°, 22.7 ± 1.9°, and 70.6 ± 1.2° (the errors provided here are standard errors of the mean, and the standard deviations are 1.5°, 3.8°, and 3.9°, respectively). The cysCNC sample is clearly more hydrophobic than both sCNC and DACNC, which is not unexpected and is further evidence that the cystamine groups are indeed present in these samples. Cystamine groups attached to the cysCNCs are rich in −CH_2_ groups, which are thought to be the main reason for the large water contact angle and hydrophobicity. This increased hydrophobicity for cysCNCs correlates with the additional −CH_2_ stretching found from the FTIR data (cf. Figure 1). For the DACNC film, the water contact angle was found to be ∼9° smaller than that for the sCNC film. To verify if all the groups of water contact angle data for sCNCs, DACNCs, and cysCNCs are significantly different, *t* tests were carried out on the means. These tests demonstrated that the mean for each data set is significantly different, with *p* < 0.05 for all samples.

### Preparation and Characterization of Gelled Pickering Emulsions

Pickering emulsions made with DACNCs were creamed rapidly within 10 min after ultrasonication. Oil droplets stabilized with DACNCs have a density lower than that of the water. This causes these oil droplets to float to the upper surface of the emulsion system and aggregate.^30^ This aggregation of the oil droplets was arrested by adding cystamine to the Pickering emulsions. The cystamine gelled the stabilized DACNC Pickering emulsions within 3 min at room temperature. During this process, the oil droplets were trapped in the gel and remained well dispersed in the aqueous system. These gelled forms of Pickering emulsions were then left to fully form at room temperature for 24 h.

The size dispersion of oil droplets and other objects within the Pickering emulsions before gelling was measured by DLS and CLSM. DACNC suspensions and the DACNC-stabilized Pickering emulsions were measured with the same DLS facility, and the resulting intensity distributions showed a clear variance by comparison. In the DACNC suspension, multiple-sized objects were detected by DLS, ranging from 12 to 615 nm (Figure S7). The intensities at 12 and 14 nm correspond roughly to the width of CNCs, which indicates the presence of isolated DACNC rods in the suspension. DACNC clusters were also detected by DLS, giving intensities at sizes ranging from 28 to 615 nm. DACNC-stabilized Pickering emulsions showed intensities from 59 to 5560 nm. It is postulated that intensities at sizes of less than 615 nm, which were also observed for DACNC suspensions, result from clusters of DACNCs and isolated CNC rods. Particles with diameters ranging from 1280 to 5560 nm detected in DACNC-stabilized Pickering emulsions are thought to be large oil droplets.

**Figure 6:**
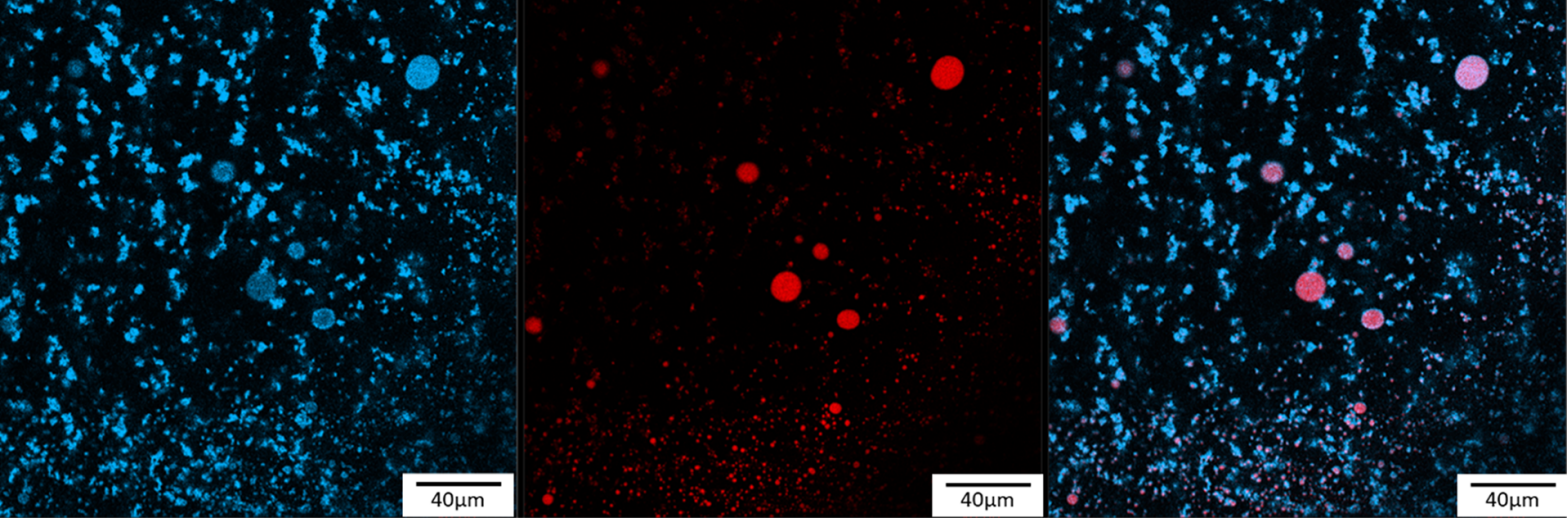
Typical CLSM images of (left) Calcofluor white-stained DACNC, (middle) Nile Red-stained linseed oil in the Pickering emulsion, and (right) an overlay of the two images.

The CLSM of Pickering emulsions showed that Calcofluor white and Nile red emulsions give fluorescence emissions at different wavelengths detected with different channels (Figure 6). In the 400–500 nm channel, the Calcofluor white-stained DACNCs were observed all over the whole Pickering emulsion forming CNC clusters of various shapes and sizes. While in the channel of Nile Red (600–700 nm), oil droplet diameters were in the range of 1.9–11 μm. The average oil droplet size was measured and found to be 2.7 ± 0.1 μm (standard error of mean with a total of 158 oil droplets measured). The measured average oil droplets’ size was similar to what has been reported for an sCNC and octylamine-CNC-stabilized linseed oil Pickering emulsion system.^30^ In the overlay image of the two channels, the red oil droplets surrounded by blue DACNCs are shown to be purple (a mix of the primary colors blue and red) with blue rings around them. Also, many blue CNC clusters remained, demonstrating that in the Pickering emulsions, oil droplets are coated by CNCs, while the remaining CNCs stay well-dispersed in the continuous water phase.

**Figure 7:**
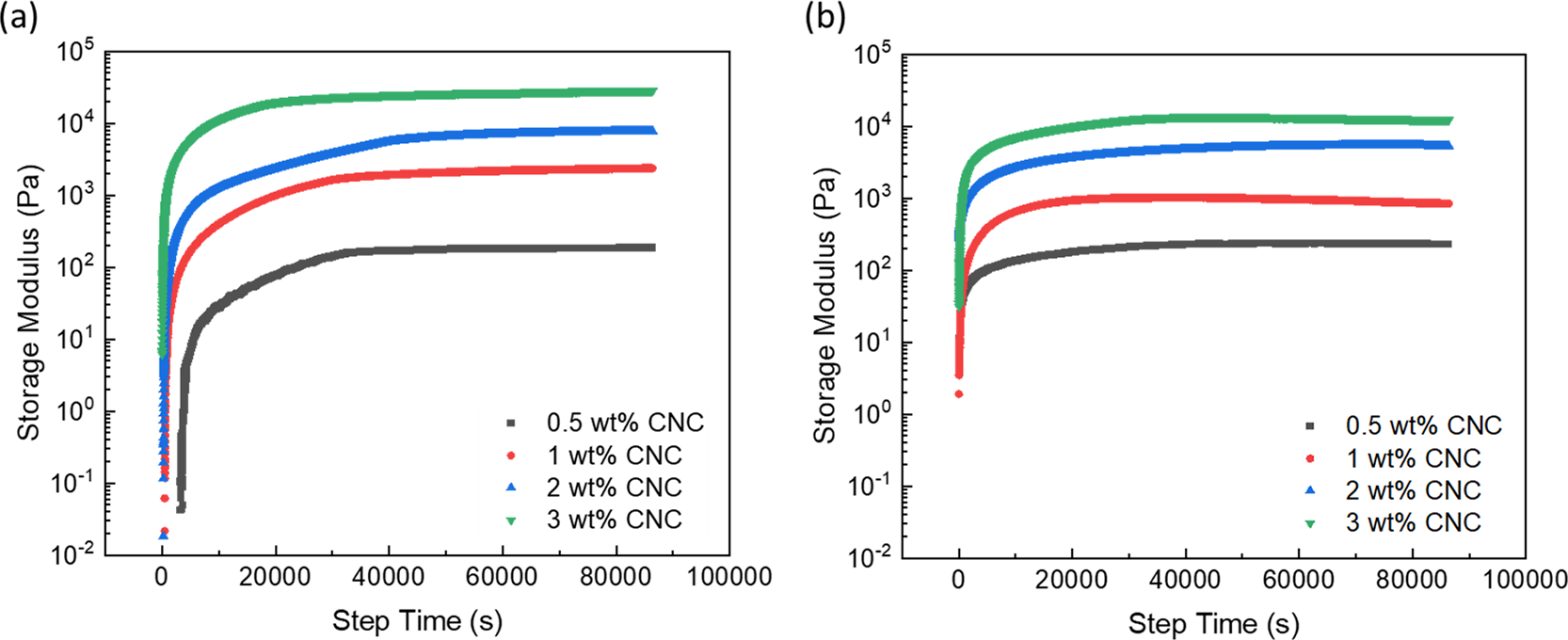
Storage modulus as a function of time for (a) gelling DACNC suspensions and (b) gelling DACNC-stabilized Pickering emulsions.

The gelling process of the Pickering emulsions was monitored by following the change in their viscoelastic properties through time sweep experiments and comparing with the same process for DACNC suspensions (Figure 7). Rapid gelling occurred within the first 20 min of the reaction for all the suspensions and emulsions, something which was also noted during sample preparation. The suspensions and Pickering emulsions were found to form “gel-like” structures during the process of adding and mixing cystamine into the systems, which was initiated after 3 min when using a magnetic rotator for mixing at room temperature. This is a key observation because the physical gel point occurs much later, but in order to slow down the gelling process and to be able to monitor this in the rheometer, the samples were cooled in an ice bath while being mixed with cystamine. The low temperature slowed the reaction between cystamine and the aldehyde groups on the DACNCs and kept the samples as a liquid before being loaded onto the serrated plate in the rheometer. The storage modulus of all samples increased to a plateau after a gelling process that occurred over approximately 11 h, while the loss modulus of all the samples was too low to be accurately measured by the rheometer. The final storage modulus of the Pickering emulsions at each concentration was lower than that of the DACNC suspensions, which indicates that the presence of the oil droplets decreases the viscoelastic properties of the gels. As the solid content of the CNC aqueous suspensions, before adding oil and the gelling took place, was kept constant, the presence of oil lowered the weight concentration of CNCs in the Pickering emulsion gel system.

**Figure 8:**
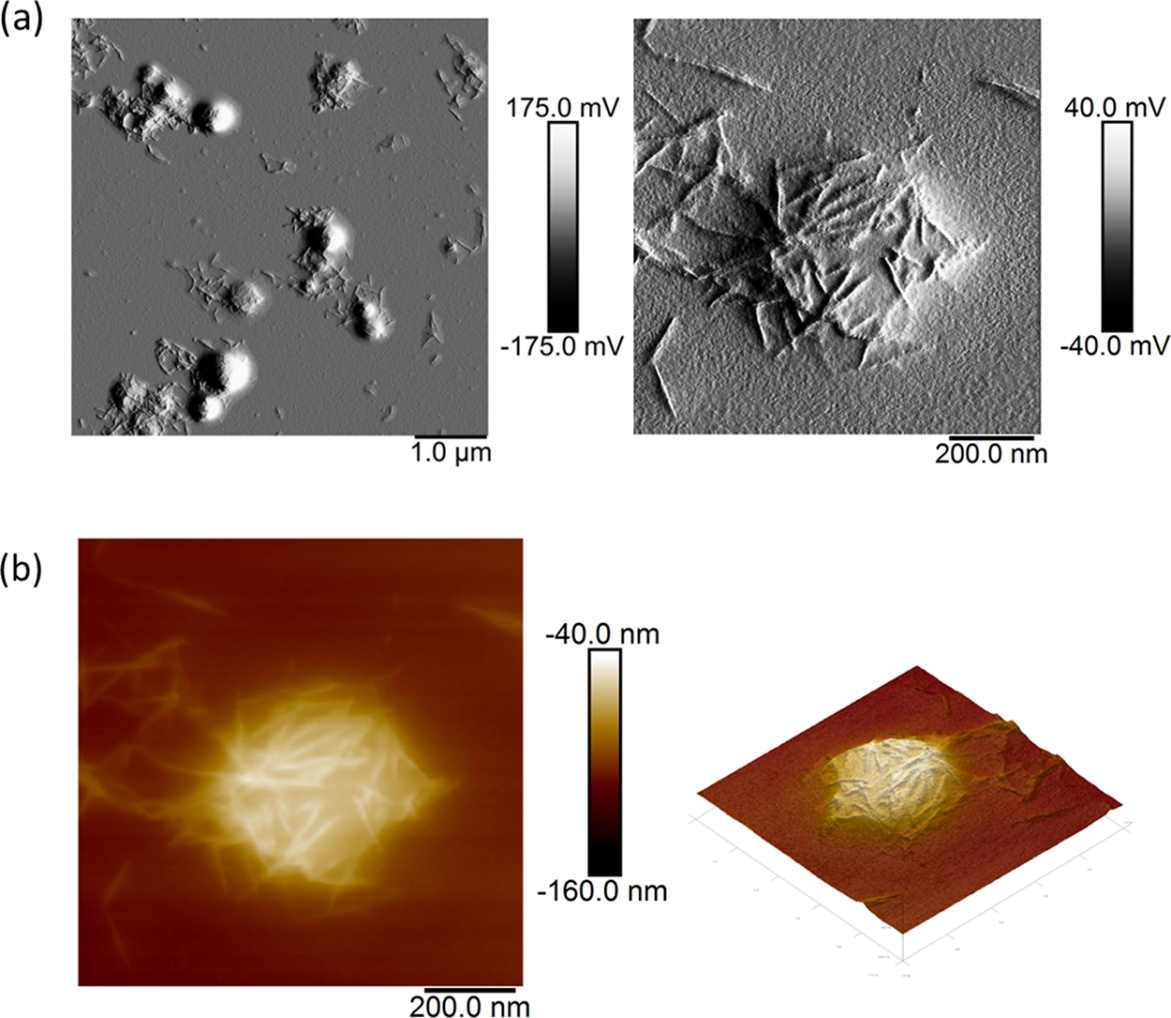
Typical AFM deflection error images of lyophilized Pickering emulsion oil droplets on silicon (a) and height images (b). The right-hand image in (a) is a close up of one of the droplets shown in the center of the main image, and for (b), it is a 3D contour plot of the same.

To get a detailed morphology of the oil droplets inside the gelled Pickering emulsions, samples were diluted with water to ∼0.5 wt % and then drop-casted onto silicon and viewed with AFM after air drying (Figure 8). The oil is thought to remain in the droplets in some locations on the silicon substrate while spreading across the surface in others. In some areas, it is noted that the CNCs remain spread in a spherically conforming arrangement, preserved from when they were surrounding an oil droplet in water. Perhaps because of the hydrophobicity of the cysCNCs, they form stable networks surrounding the droplets, and for the most part, they retain the hydrophobic cystamine groups within the oil (Figure S8). Their propensity to adhere to each other has then meant that they formed strong networks.

**Figure 9:**
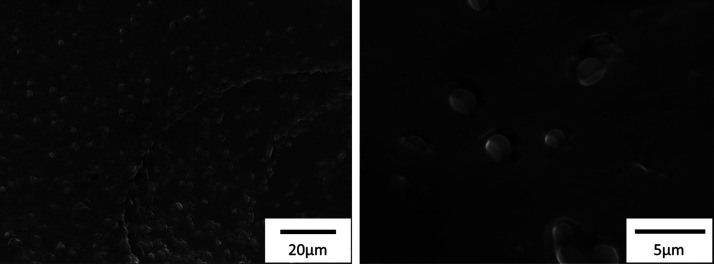
Typical SEM images of freeze-dried gelled Pickering emulsions; micrograph showing the size range of droplets and their dispersion (left) and a higher magnification image of droplets (right).

The gelled Pickering emulsions were frozen with liquid nitrogen and lyophilized for 3 days to obtain freeze-dried samples. Freeze-dried samples were found to be all white due to light scattering which was also seen for the lyophilized CNC samples. No leaking of any yellow-colored linseed oil at the surface of the samples or in the chamber of the freeze-drier was observed, indicating that cross-linked CNCs maintained the network structure even without water. Oil droplets were observed to be well dispersed inside the freeze-dried samples from SEM images (Figure 9). CysCNCs are presumed to assist the oil droplets in maintaining their spherical shape and maintaining their distance from one another after lyophilization. The average diameter of the oil droplets in the freeze-dried gelled Pickering emulsions was measured to be 2.2 ± 0.04 μm (standard error of the mean with a total of 151 oil droplets counted). The average oil droplet diameter measured in the freeze-dried gelled Pickering emulsion samples was smaller than the size determined by CLSM (2.7 ± 0.1 μm) before gelling. It is thought that the difference between the measured oil droplet size might be caused by the cross-linking of DACNCs at the oil droplet surfaces during the gelling process and the shrinking of oil droplets during the freeze-drying process. The oil droplets in the freeze-dried samples were all embedded in the CNC networks, which might also cause the measured size from the SEM images to be smaller than other methods.

For the CNC-covered linseed oil droplets, it was assumed that the linseed oil density ρ_1_ = 0.93 g cm^–3^, the CNC density ρ_2_ = 1.5 g cm^–3^, and the measured oil droplet radius *R* = 1.1 μm. Assuming that all the DACNCs were adsorbed to the oil droplet’s surfaces (*M*_2_/*M*_1_ = 20%), the predicted CNC shell thickness was found, using eq 1, to be ∼44 nm. Such a large shell thickness would mean that CNCs would need to form multiple layers around each oil droplet. It is thought that the CNCs only form a single layer of shell around each oil droplet, so then taking the average shell thickness *x* to be 8.2 nm (as seen from AFM and TEM images) we can calculate the mass ratio of CNCs (*M*_2_/*M*_1_) using eq 2. This was found to be 3.6%. However, given that the mass ratio of CNC added in the Pickering emulsion and linseed oil was 20%, a large proportion of the added CNCs were clearly not adsorbed to the water–oil interfaces, which is also noted from the CLSM (Figure 6) and AFM (Figure 8) images. This suggests that there is much more to be done to optimize the adsorption of CNCs at oil–water interfaces and make the best use of the solid phase in a Pickering emulsion.

### Fabrication and Characterization of Self-Healing Coatings

**Figure 10:**
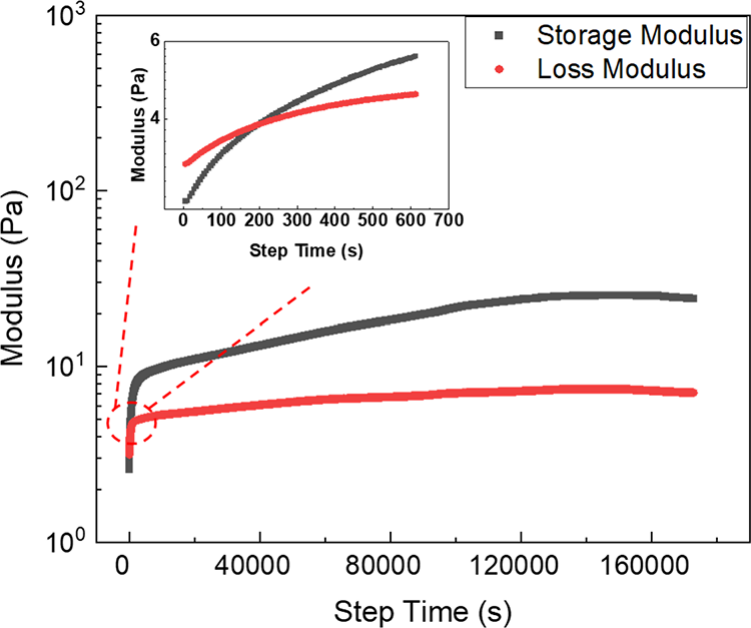
Storage and loss modulus over time for a gelling Pickering emulsion mixture and a PVA solution mixture.

Well-dispersed linseed oil droplets were utilized as an in situ formed self-healing agent and were mixed with a 10% PVA solution. To ensure good dispersion of oil droplets in the PVA solution and prevent their aggregation during the long air-drying process (over 24 h), the DACNC-stabilized Pickering emulsions were first mixed with the PVA solution using magnetic stirring and then agitated in an ultrasonic bath. Following this, gelling of the system was initiated by adding cystamine to this mixture. The gelling process of the DACNC-stabilized Pickering emulsion and PVA solution mixture was again monitored, as before, with a rheometer (Figure 10). Similar to the process in DACNC suspensions and DACNC-stabilized Pickering emulsions, the gelling was rapid within the first 20 min and gradually slowed down until the reaction finished. With a 10% PVA solution mixed with the Pickering emulsion, the storage modulus and loss modulus of the sample were high enough to be measured by the rheometer, and both values rose to a plateau. This whole process took over 44 h, which was longer than for the previous samples shown in Figure 8. It is likely that this process is limited by diffusion. A clear gel point at which the storage modulus equaled and surpassed the loss modulus of the sample was found at around 200 s. The gelled PVA and Pickering emulsion mixtures were then drop-casted onto glass slides and air-dried into coatings. Compared with a one-step fabrication process of the coating, in previous research, self-healing epoxy coatings were made from first synthesizing urea–formaldehyde capsules with in situ polymerization of surfactant-emulsified linseed oil emulsions (followed by washing and drying) and then mixing capsules with epoxy resin and polymerizing them again.^37,38^

**Figure 11:**
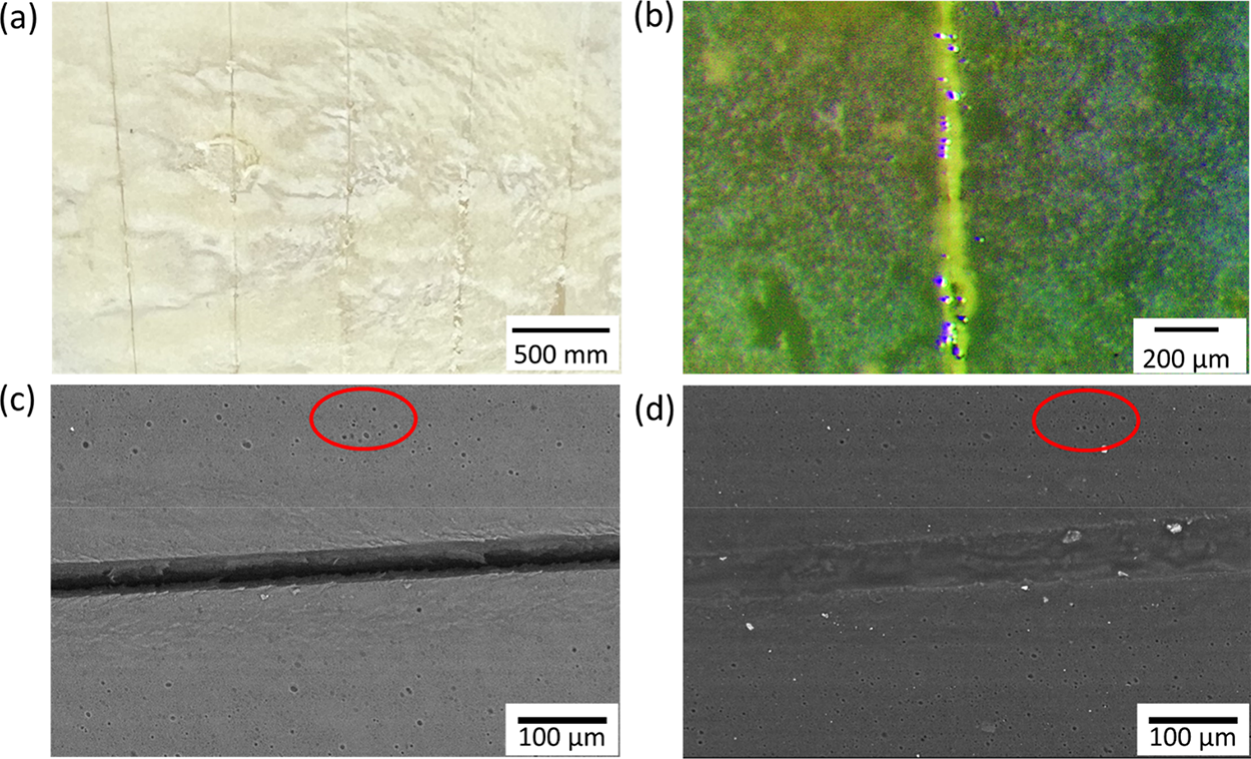
Typical (a) photograph of a scratched composite coating, (b) optical microscope of a healed scratch, and (c) backscattered SEM images of a scratch before (left) and after (right) healing.

The epoxy coating system-embedded urea–formaldehyde-encapsulated linseed oil droplets in epoxy coatings were applied to mild steel panels and were scratched before leaving them to heal at room temperature for 24 h.^38^ Their corrosion test showed that linseed oil at the scratches can inhibit the corrosion of the steel substrate when the scratched samples were exposed to sodium chloride aqueous solution; however, the oxidation and solidification of linseed oil were not clearly shown in their research.^38^ In our experiments, the self-healing ability of the composite coatings was tested by making scratches with a scalpel. After a series of scratches were made on the coating, the samples were imaged using SEM under low vacuum before and after healing as a demonstration of the self-healing effect. It was reported that for the oxidation of linseed oil at 80 °C, to form about 60% of insoluble polymers the linseed oil should react with oxygen in the air for 16 h, which was 40 times faster compared with the oxidation at room temperature.^35^ To ensure a good healing effect on our coatings, no conductive coating was applied to the sample for the SEM to not confuse with the cracking of the sample, and the sample was then placed in an oven at 80 °C for 16 h for healing. After heating in the oven, leaked linseed oil within the scratches oxidized and hardened into a solid material, which is clearly observed from the photograph and the optical microscopic image shown in Figure 11a,b. The red circles in the typical SEM images shown in Figure 11c highlight the presence of air bubbles on the coating surface, with their location and arrangement demonstrating that the scratch area was imaged before and after (Figure 11c,d) healing. Linseed oil clearly fills the gap and heals the scratch by hardening. The scratches were also demonstrated to heal gradually at room temperature during a period of 21 days; the healing process was viewed with SEM under low vacuum every 7 days (Figures S9 and S10).

## Conclusions

We have studied the influence of cross-linking CNCs with cystamine groups through periodate oxidation and Schiff base reaction on the thermostability and surface activity of dried CNCs. Cystamine groups were demonstrated to decelerate the degradation of cysCNCs compared to that of DACNCs, which increased the hydrophobicity of CNCs. Pickering emulsions were demonstrated by breaking linseed oil into droplets in DACNC suspensions and the autoadsorption of a small part of DACNCs to the water–oil interfaces forming a single layer of the CNC network at their surfaces. The vast majority of DACNCs were found to be dispersed in the continuous water phase, which were then cross-linked, either by imide bonds or by physical interactions, into CNC networks after adding cystamine. The presence of cystamine was found to gel DACNC Pickering emulsions, and their mixture with PVA solutions occurs rapidly before the creaming of oil droplets happens. This is thought to keep the oil droplets well dispersed. It was found that drop-cast gelled Pickering emulsions and PVA composite coatings exhibited self-healing abilities for the scratches made on the coatings. These gelled Pickering emulsions are thought to be a new type of stable form, and could have applications beyond what we report here to provide encapsulation in food, cosmetics, and paint-based systems.

## Data Availability

Data generated from this work is freely available from the University of Bristol’s repository at https://data.bris.ac.uk/data/.

## Supporting Information Available

Typical conductivity titration results of sCNC and DACNC; typical XPS spectrum (left) and the fitting of the region of this spectrum with two sulfur 2p peaks (right) for cysCNCs; solution-state 1H NMR spectrum of cysCNC, with peak annotation for the CNC backbone, methylene groups in cystamine and DMSO; 1H DOSY NMR spectrum of cysCNC; TGA (left) and derivative data (right) for sCNC, DACNC, and cysCNC in nitrogen; typical TEM images of crosslinked cysCNCs; typical intensity weighted size distribution of a DACNC suspension and a Pickering emulsion stabilized with DACNCs measured by DLS; AFM height images (a), adhesion images (b), peak force error (c), and surface topography (d) of lyophilized Pickering emulsion oil droplets on silicon at selected sample voltages; scratch automatic width change at room temperature in 21 days; and typical SEM images of scratch healed under room temperature (PDF)

## Abbreviations

AFM: atomic force microscopy
CLSM: confocal laser scanning microscopy
CNC: cellulose nanocrystal
CNM: cellulose nanomaterial
cysCNC: cystamine-cross-linked cellulose nanocrystal
DAC: dialdehyde cellulose
DACNC: dialdehyde cellulose nanocrystal
DI: water deionized water
DLS: dynamic light scattering
FTIR: Fourier-transform infrared spectroscopy
PVA: poly(vinyl alcohol)
SEM: scanning electron microscopy
TEM: transmission electron microscopy
TGA: thermogravimetric analysis
WCA: water contact angle test
XPS: X-ray photoelectron spectroscopy
