# Supplementary material for: Self-Healing Composite Coating Fabricated with a Cystamine Cross-Linked Cellulose Nanocrystal-Stabilized Pickering Emulsion
Source: Biomacromolecules. 2024 Jan 25;25(2):715–28. doi: 10.1021/acs.biomac.3c00915 (PMC10865351; doi:10.1021/acs.biomac.3c00915)
Supplement: Supplementary file 1 — bm3c00915_si_001.pdf [file bm3c00915_si_001.pdf]

Supplementary Information

Self-healing composite coating fabricated with a cystamine crosslinked cellulose nanocrystal stabilized Pickering emulsion

Guofan Xu[1], Amaka J. Onyianta[1], Jean-Charles Eloi[2], Robert L. Harniman[2], Jude Laverock[2], Ian Bond[1], Onajite Abafe Diejomaoh[1], Todor T. Koev[3], Yaroslav Z. Khimyak[3], Stephen J. Eichhorn[1]*

1. Bristol Composites Institute, School of Civil, Aerospace and Design Engineering (CADE), University of Bristol, University Walk, Bristol, BS8 1TR, UK.

2. School of Chemistry, University of Bristol, Bristol, BS8 1TS, UK.

3. School of Pharmacy, University of East Anglia, Norwich Research Park, NR4 7TJ, UK.

*Email: s.j.eichhorn@bristol.ac.uk

	Mean	Standard Deviation	Standard Error from Mean
DACNC length /nm	113.7	32.6	4.86
DACNC width /nm	8.0	1.6	0.20
CysCNC length /nm	111.8	36.3	6.52
CysCNC width /nm	8.2	1.9	0.19

Table S1. Average dimensions of DACNC and cysCNC with standard deviations and errors from mean. Data taken from TEM images of CNCs.

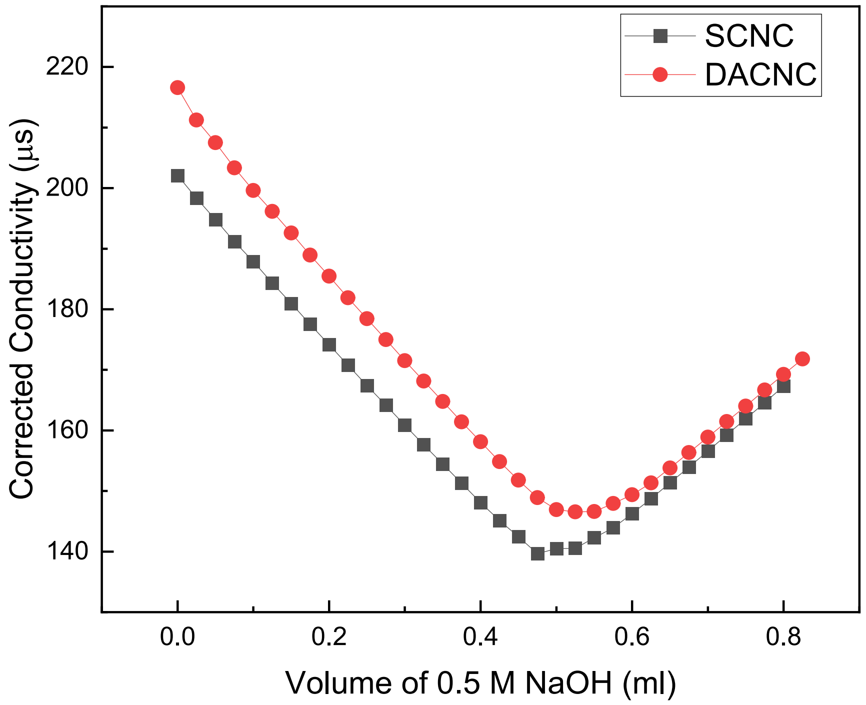

Figure S1. Typical conductivity titration results of sCNC and DACNC.

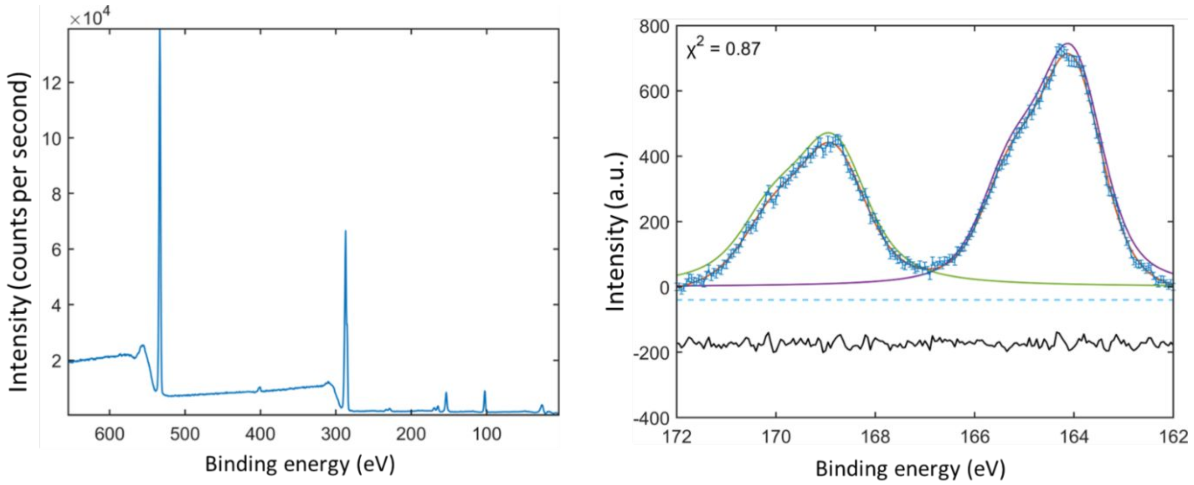

Figure S2. A typical XPS spectrum (left) and the fitting of the region of this spectrum with two sulfur 2p peaks (right) for cysCNCs.

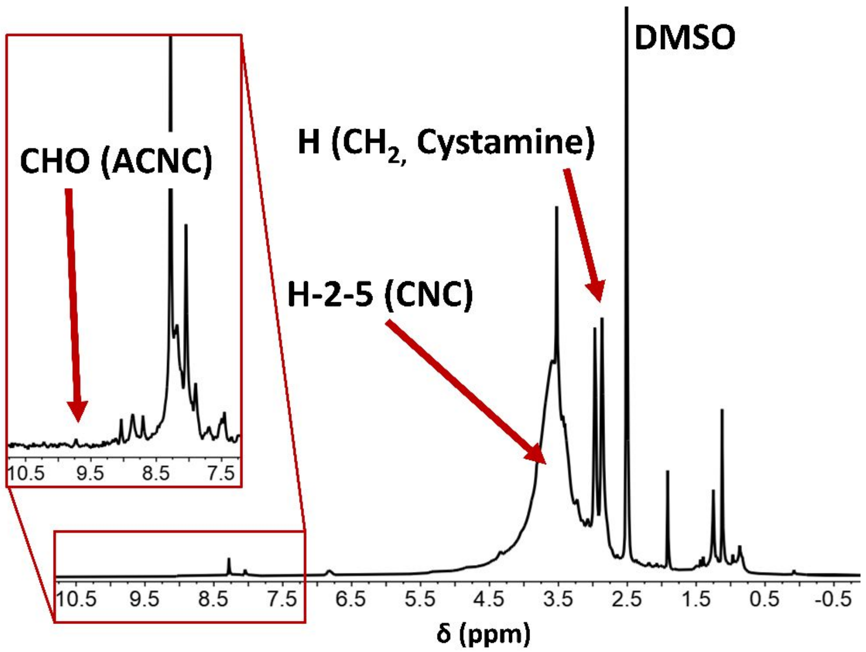

Figure S3. Solution-state ^{1}H NMR spectrum of cysCNC, with peak annotation for the CNC backbone, methylene groups in cystamine and DMSO. Inlay showing a small peak (ca. 9.6 ppm), characteristic of an aldehyde group.

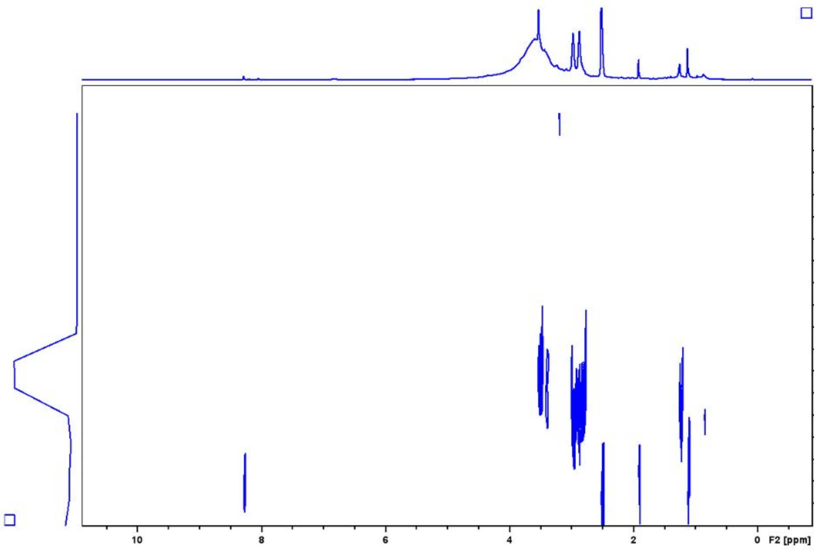

Figure S4. ^{1}H DOSY NMR spectrum of cysCNC.

Fitting self-diffusion coefficients from ^{1}H DOSY NMR. CNC and cystamine ^{1}H peaks (3.2-4.0 ppm, and ca. 2.8-3.0 ppm, respectively) were integrated and their signal decay as a function of gradient strength was fitted according to Equation S1, using Bruker TopSpin v4.1.4's Dynamics Centre

$$I = I_0 exp\,(-D\gamma^2 g^2 \delta^2 \left(\Delta - \frac{2\delta}{3} - \frac{\tau}{2}\right)) \qquad \text{Equation S1}$$

where I and I_0 are the observed and reference peak intensity, D is the self-diffusion coefficient, γ is the gyromagnetic ratio of the observed nucleus, g is the gradient strength, δ is the length of the gradient, Δ is the diffusion time, and τ is the gradient recovery period.

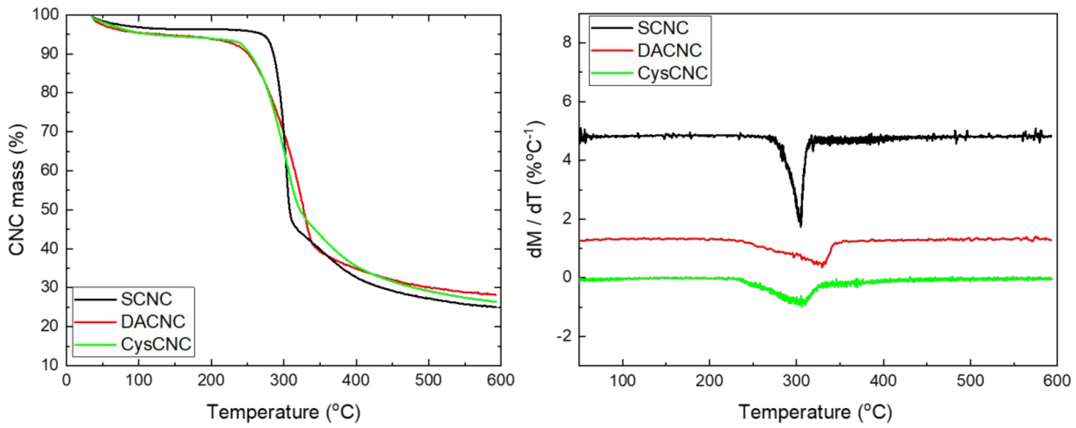

Figure S5. Thermogravimetric (TGA) analysis (left) and derivative data (right) for sCNC, DACNC and cysCNC in nitrogen.

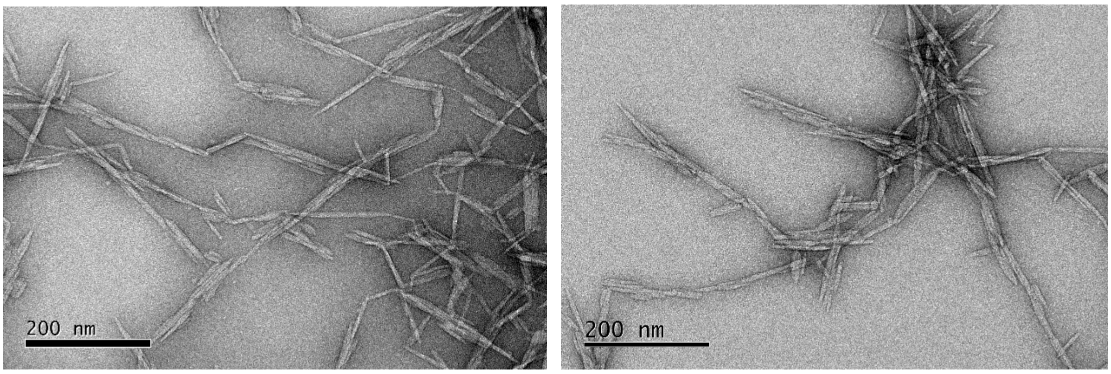

Figure S6. Typical TEM images of crosslinked cysCNCs.

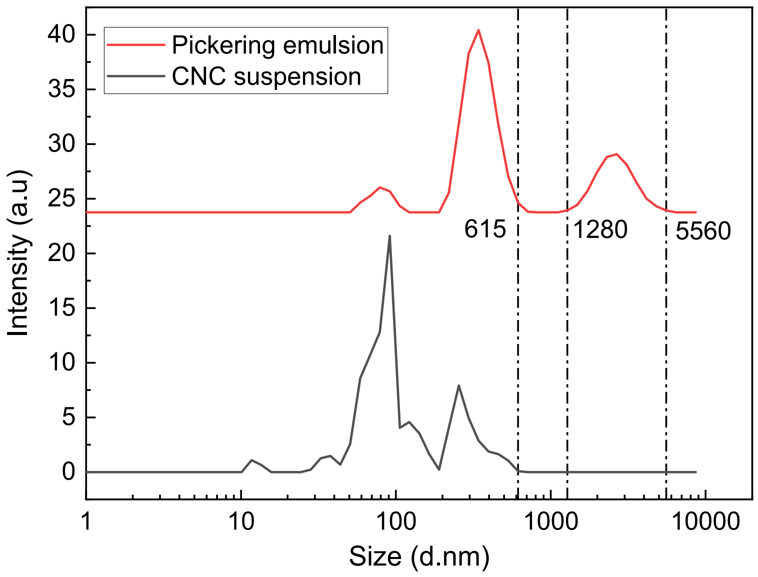

Figure S7. Typical intensity weighted size distribution of a DACNC suspension and a Pickering emulsion stabilized with DACNCs measured by Dynamic Light Scattering (DLS).

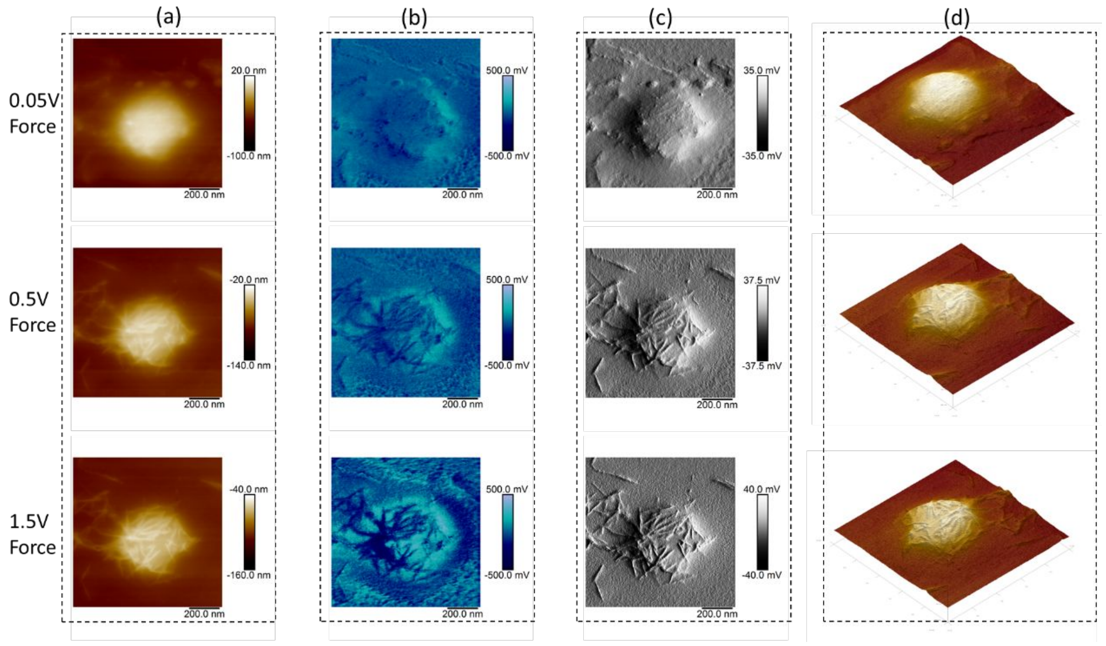

Figure S8. AFM height images (a), adhesion images (b), peak force error (c) and surface topography (d) of lyophilized Pickering emulsion oil droplets on silicon at selected sample voltages.

AFM images of a monolayer gelled Pickering emulsion were collected at setpoints of 0.05 V, 0.5 V and 1.5 V. The CNC mesh on the oil droplets became clearer with an increase in the setpoint, indicating that some portion of the CNC meshes were immersed inside the oil droplets. The nominal spring constant and tip radius of the cantilevers used was 0.4 N/m and 2nm respectively. With the typical deflection sensitivity of the system. By multiplying the setpoint voltages by a conversion factor of 12nN/V one can covert to an applied force; 600pN for 0.05V and 18nN for 1.5V. It is these forces that penetrate through the sample yielding images of the CNCs as the voltage is increased.

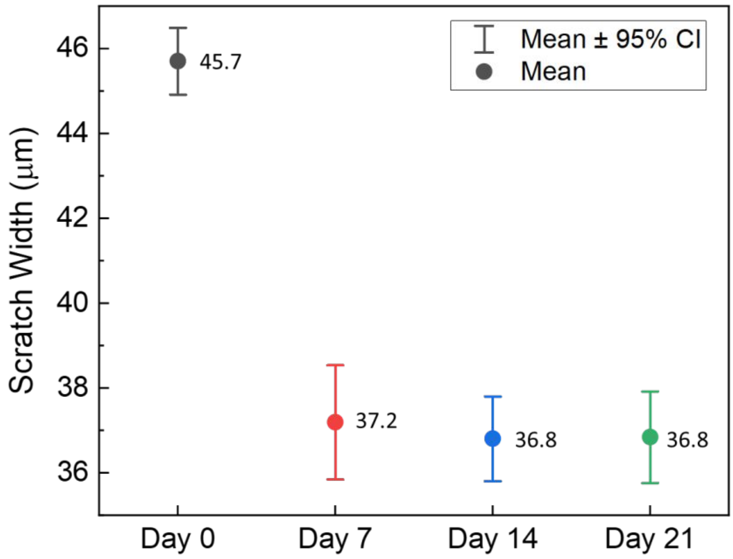

Figure S9. Scratch automatic width change at room temperature in 21 days.

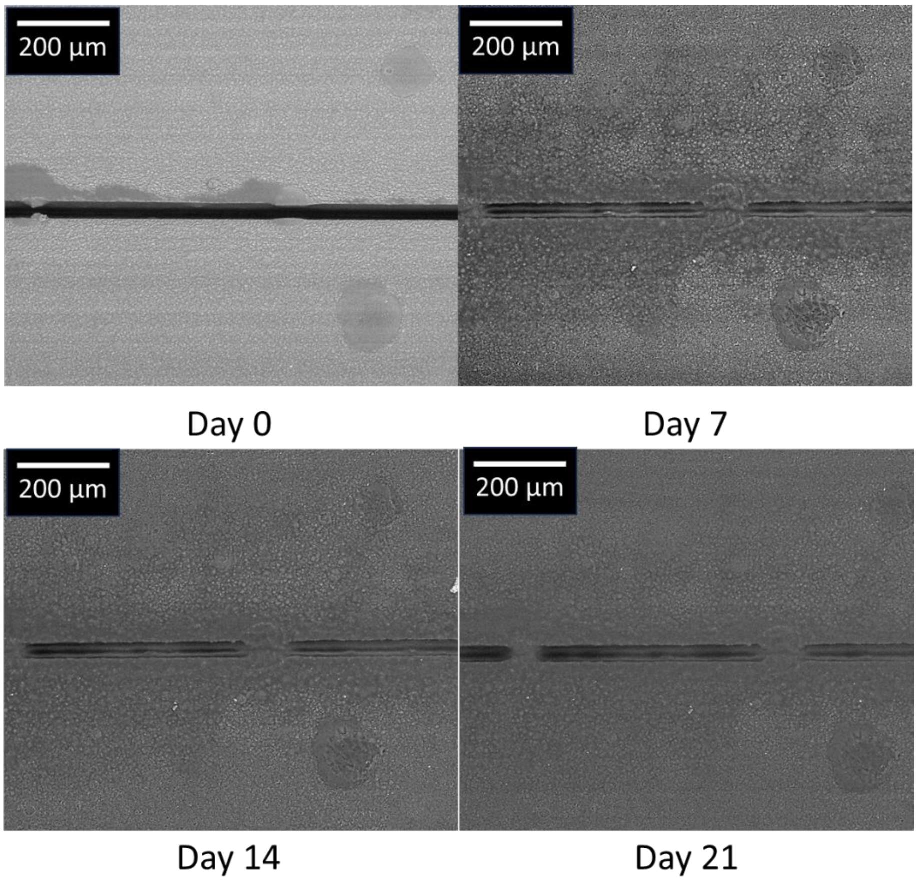

Figure S10. Typical SEM images of scratch healed under room temperature.

After scratching the coating with a scalpel, the coating was left at room temperature for healing. The scratch was viewed with SEM every 7 days in low vacuum without any silver (Ag) coating. A clear narrowing of the scratch width was observed after exposing the scratch to the air for 7 days, while the scratch width then stayed almost constant during the rest of the time. This was thought to be caused by the elasticity of the PVA coating.

The leak of oil was seen in the day 0 SEM image at the edge of the scratches as the cutting of scalpel went through and break the oil droplets. The scratch was then filled with oxidized linseed oil in the following days.
